# Supplementary material for: Influenza A Virus Infection Reactivates Human Endogenous Retroviruses Associated with Modulation of Antiviral Immunity
Source: Viruses. 2022 Jul 21;14(7):1591. doi: 10.3390/v14071591 (PMC9320126; doi:10.3390/v14071591)
Supplement: Supplementary file 1 [file viruses-14-01591-s001.zip › MotifAnalysis.zip!/MotifOutput_SC35M/homerResults/motif34.similar.html]

motif34

## Information for motif34

A
T
C
G
A
G
T
C
A
C
G
T
A
C
G
T
A
C
G
T
C
G
A
T
C
G
T
A
C
G
T
A
C
G
A
T
A
C
T
G
  
Reverse Opposite:  

A
G
T
C
C
G
T
A
A
C
G
T
A
C
G
T
C
G
T
A
C
G
T
A
C
G
T
A
C
G
T
A
A
C
T
G
A
T
G
C
  

|  |  |
| --- | --- |
| p-value: | 1e-3 |
| log p-value: | -8.666e+00 |
| Information Content per bp: | 1.940 |
| Number of Target Sequences with motif | 3.0 |
| Percentage of Target Sequences with motif | 7.32% |
| Number of Background Sequences with motif | 122.6 |
| Percentage of Background Sequences with motif | 0.26% |
| Average Position of motif in Targets | 118.4 +/- 31.1bp |
| Average Position of motif in Background | 90.6 +/- 66.2bp |
| Strand Bias (log2 ratio + to - strand density) | -0.6 |
| Multiplicity (# of sites on avg that occur together) | 1.67 |
| Motif File: | file (matrix) reverse opposite |

### Similar de novo motifs found

|  |  |  |  |  |  |  |  |
| --- | --- | --- | --- | --- | --- | --- | --- |
| Rank | Match Score | Redundant Motif | P-value | log P-value | % of Targets | % of Background | Motif file |
